# Supplementary material for: Continuous evaluation of cost-to-go for flexible reaching control and online decisions
Source: PLoS Comput Biol. 2023 Sep 27;19(9):e1011493. doi: 10.1371/journal.pcbi.1011493 (PMC10561875; doi:10.1371/journal.pcbi.1011493)
Supplement: S1 Text — (DOCX) [file pcbi.1011493.s001.docx]

**S1 Text. Simulations in presence of multiplicative noise**

In this section, we show that our model generalizes to the presence of signal-dependent noise, which was not addressed in the main sections of the paper for simplicity. We consider a dynamical system with additive Gaussian noise and multiplicative noise affecting the control vector and feedback signals captured by the following equations :

$$x_{t+1}=A x_{t}+B u_{t}+\xi_{t}+ \sum_{i=1}^{n_{c}} \epsilon_{i,t}C_{i}u_{t}$$

$$y_{t}=H \hat{x}_{t}+\omega_{t} + \sum_{i=1}^{n_{d}} \delta_{i,t}D_{i}x_{t}$$

Where $\xi_{t}\sim N\left( 0,\Omega_{\xi} \right)$ and $\omega_{t}\sim N\left( 0,\Omega_{\omega} \right)$ are the motor and sensory additive gaussian noises, the terms $C_{i}$ and $D_{i}$ are scaling matrices used in the definition of the signal dependent noise, and $\epsilon_{i,t}\sim N\left( 0,1 \right)$, $\delta_{i,t}\sim N(0,1)$ are Gaussian random variables. We can use the same state estimation and cost function as in the main paper, respectively equations (6) and (7).

By defining the estimation error as the difference between the true state of the system and its estimate $e_{t}≝x_{t}- \hat{x}_{t}$, the expression for the cost-to-go under the optimal policy (see (1) for the detailed derivation) becomes:

$$v_{t}=x_{t}^{T}S_{t}^{x}x_{t}+e_{t}^{T}S_{t}^{e}e_{t}+s_{t}$$

The second term, which captures the cost related to the estimation error, is the only difference with equation (19) and follows from the introduction of signal-dependent noise. The impact of target reward, which was modeled using the term $s_{t}$ can also be added to the problem featuring signal-dependent noise by offsetting the same term, independent of the term linked to the estimation error. Therefore, the properties presented in the paper are still valid modulo the increase in variability. To highlight that the main features of the paper could be reproduced in this context, we added Fig A with simulations of the effect of a change in target structure, and the dependency of decision time and frequency of target switches on the reward landscape and perturbation amplitude (implemented as in (1,2)).


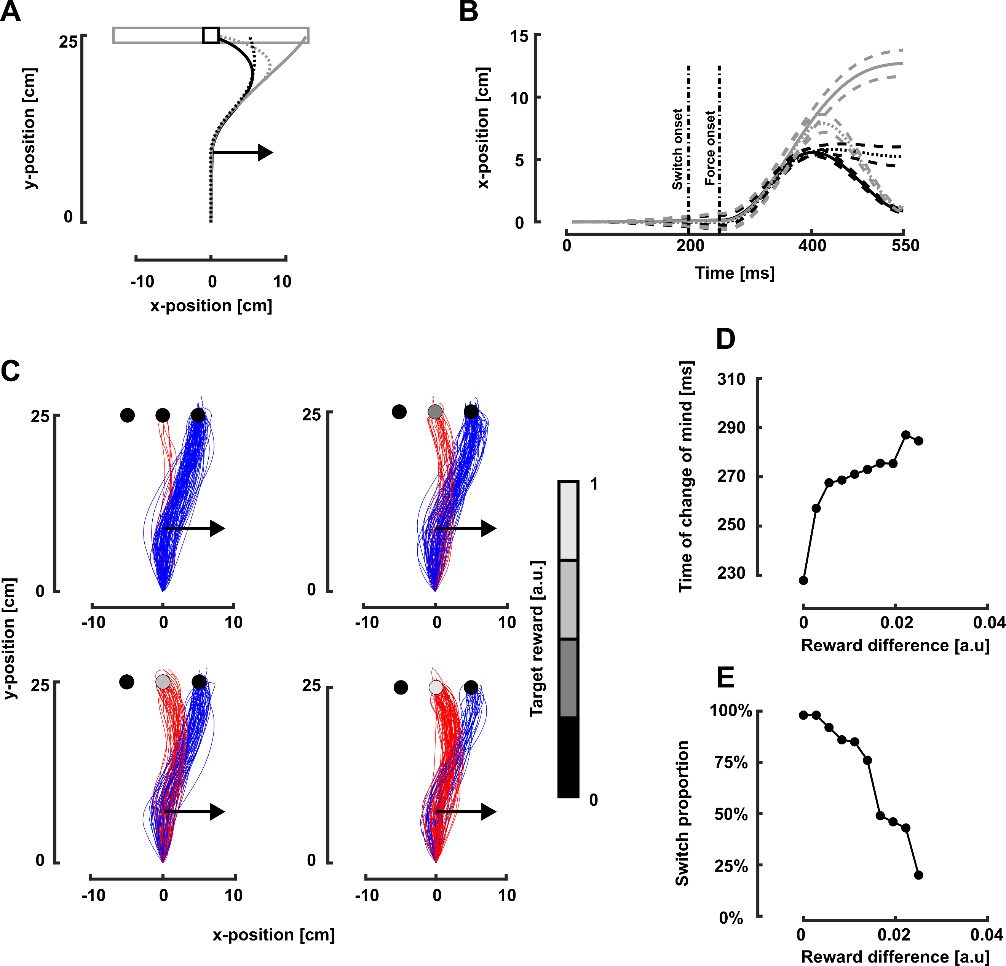


Fig A. Reproduction of the main results in presence of signal-dependent noise. **A** Average reaching behavior to a square (full line black), rectangle (full line gray), square-to-rectangle (dashed line black), and rectangle-to-square (dashed line gray) targets in presence of a leftward mechanical perturbation. **B** Mean and standard deviation of the lateral deviation induced by the mechanical perturbation for the square (full line black), rectangle (full line gray), square-to-rectangle (dashed line black), and rectangle-to-square (dashed line gray) conditions. **C** Individual reach trajectories in presence of lateral perturbation (schematized by the black arrow) in the decision task where targets with different rewards were presented. Red and blue traces highlight movements reaching the central and right target respectively. The relative reward of each target is captured by their color (darker colors represent less rewarding targets). **D** Representation of the mean of the time at which the decision to switch target occurred as a function of reward difference (larger values correspond to larger relative reward for the central target) .**E** Proportion of trial switching to the lateral target as a function of the reward difference. Large reward differences correspond to a larger reward for the central target compared to the lateral ones.

**References**

1. Crevecoeur F, Sepulchre RJ, Thonnard JL, Lefèvre P. Improving the state estimation for optimal control of stochastic processes subject to multiplicative noise. Automatica. 2011;47(3):591‑6.

2. Todorov E. Stochastic Optimal Control and Estimation Methods Adapted to the Noise Characteristics of the Sensorimotor System. Neural Comput. 2005;17(5):1084‑108.
