# Supplementary material for: Continuous evaluation of cost-to-go for flexible reaching control and online decisions
Source: PLoS Comput Biol. 2023 Sep 27;19(9):e1011493. doi: 10.1371/journal.pcbi.1011493 (PMC10561875; doi:10.1371/journal.pcbi.1011493)
Supplement: Attachment — Submitted filename: ResponseReviewers.pdf [file pcbi.1011493.s002.pdf]

We would like to thank the Reviewers and the Editors for their in-depth assessment of our work and for raising the important points below. These helped us to significantly improve the paper. Please find below a point-to-point response to the comments that were made along with the references to the modifications of the text. The sections of the manuscript that were changed are included in our responses in *italic* and the specific changes are written in red.

As with all papers reviewed by the journal, your manuscript was reviewed by members of the editorial board and by several independent reviewers. The reviewers were generally positive about the paper and the ability of the model to account for experimental observations. However, the reviewers raised several concerns. Most importantly, the reviewers felt that there was insufficient discussion of conceptually related preceding literature. In particular, recent papers from Vassileios Chritopoulos (e.g. Enachescu et al., PLOS CB 2021). I would also add a paper by Emanuel Todorov ("Compositionality of optimal control laws", NIPS 2009). These papers both propose models in which a variable analogous to the cost-to-go is used to arbitrate between multiple policies during the course of a movement. (see also Rigoux and Guigon, PLOS CB 2012). In addition, the reviewers raised some issues about the formulation of the model (including whether model predictive control is the appropriate framework) and whether or not alternative hypotheses could be adequately ruled out.

We are grateful for the opportunity to explain our work in a broader context. Specifically, we discussed how our work conceptually compares to preceding literature, mostly the go-before-you-know paradigms in which the interplay between motor control and decision making was previously studied. We emphasized that the tasks we studied in the present paper differed from these previous tasks as they did not involve any uncertainty concerning the correct target to reach within each movement. We also detailed our model implementation and how it compares with other proposed models that leveraged similar variables during movement.

Reviewer 1

Here the authors propose a computational model that uses a higher-level decision-making controller that chooses at each time point the minimal cost-to-go from several parallel plans. The cost on accuracy is manipulated to accommodate target width, target size rate of change, and reward. Using this model, they can replicate hand trajectories from a number of previously published results. The paper was an enjoyable read, the model and simulations were well described, and the manuscript is worthy of publication. Below are mostly high-level comments to further improve the paper. Can the authors please respond in a point-by-point manner and refer to line numbers with manuscript changes.

Specific Concerns

1. This first point is probably the most important to address in the paper. Since its introduction to the field, optimal feedback control has been one of the most powerful frameworks to understand human behavior. What has been powerful about this approach is that a lot of behaviour emerges from the model. Here, however, the authors manipulate the cost function differently for each new experimental condition in an ad hoc fashion---rather than all the behaviour emerging from the same model, with parameters and cost function held constant, given task demands. In other words, applying a new cost function for each experimental condition in an ad hoc manner is less powerful than having behavior emerge from the same model. Can the authors please include this as a limitation within the discussion.

We understand the Reviewer’s concern and discuss that point in more detail in the revised version of the manuscript. A single parametrization of the optimal feedback control model can indeed result in different behavioral realizations. Reaching behaviors in presence of perturbations (visual or mechanical) and the selective

corrections for the effect of noise can be generated using the same cost-function as the one used to generate unperturbed movements (Todorov & Jordan, 2002). However, alterations of the target structure (Knill et al., 2011; Nashed et al., 2012), adaptation mechanisms to velocity-dependent force fields (Crevecoeur et al., 2020; Ikegami et al., 2021) or multiple potential targets (Nashed et al., 2014) necessitate adjustments of the controller, either by changing the cost-function to accommodate specific task requirement, or by changing the model parameters to represent adaptation. The set of tasks that we considered in the present study fall in the category where sudden changes of goal or target structure requires considering the respective cost-function of each configuration or option. The specific contribution of this paper is to demonstrate that the within-movement corrections observed experimentally can be predicted by updating the control policy online thanks to a continuous calculation of the cost-to-go. We clarified this point in the introduction (**lines 86-87)** and discussion sections (**lines 415-417**).

"Even though the OFC framework has been extensively used to explain flexible feedback control (2,29–32), it was mainly applied to static environments: that is, cost parameters have been typically considered fixed during a given trial. Therefore, a mechanism able to respond to these sudden changes in task parameters as observed in previous experimental studies is required."

"To accommodate the experimental observations made about the dependency of these changes, we proposed an extended implementation of the OFC framework combined with a decision module based on a continuous observation of the target structure and an estimation of the cost-to-go function. The addition of this decision module to the native OFC implementation is necessary as it is often assumed that the cost function, and therefore the feedback gains, are fixed during movement, which was not compatible with the experimental results captured in this study."

2. The intersection of sensorimotor control and decision-making has been modelled previously, but these are noticeably not discussed in the paper and worthy of a discussion paragraph. Some of these citations are below.

• Haith, A. M., Huberdeau, D. M., & Krakauer, J. W. (2015). Hedging your bets: intermediate movements as optimal behavior in the context of an incomplete decision. PLoS computational biology, 11(3), e1004171.

• Christopoulos, V., Bonaiuto, J., & Andersen, R. A. (2015). A biologically plausible computational theory for value integration and action selection in decisions with competing alternatives. PLoS computational biology, 11(3), e1004104.

• Enachescu, V., Schrater, P., Schaal, S., & Christopoulos, V. (2021). Action planning and control under uncertainty emerge through a desirability-driven competition between parallel encoding motor plans. PLoS computational biology, 17(10), e1009429.

Further, these papers and others noted below, suggest competing hypotheses as to whether the sensorimotor system uses a single flexible plan or averages parallel plans. Here the authors provide a slightly different idea. That the nervous system evaluates all parallel plans but can flexibly switch to the plan with the current lowest cost-to-go. Two things to address: 1.) Can the authors please include in the discussion on how their model fits in the literature with other models and hypotheses in the field. 2.) Can the model predict the results of Chapman et al. (2010) or Wong, A. L., & Haith, A. M. (2017) in a go-before-you-know paradigm when the cost-to-go for each potential target is the same.

• Wong, A. L., & Haith, A. M. (2017). Motor planning flexibly optimizes performance under uncertainty about task goals. Nature communications, 8(1), 14624.

• Chapman, C. S., Gallivan, J. P., Wood, D. K., Milne, J. L., Culham, J. C., & Goodale, M. A. (2010). Reaching for the unknown: multiple target encoding and real-time decision-making in a rapid reach task. Cognition, 116(2), 168-176.

• Gallivan, J. P., Logan, L., Wolpert, D. M., & Flanagan, J. R. (2016). Parallel specification of competing sensorimotor control policies for alternative action options. Nature neuroscience, 19(2), 320.

• Alhussein, L., & Smith, M. A. (2021). Motor planning under uncertainty. Elife, 10, e67019.)

We agree that the intersection of sensorimotor control and decision-making was previously discussed in the series of studies mentioned by the Reviewer, but we want to emphasize an important difference between

these tasks and the ones studied in the present work. These previous studies used a *go-before-you-know* paradigm, in which the subject initiated their movements before knowing which of the presented target was the one that would be cued later, thus this paradigm involves planning uncertainty but no online decision to change goal as the correct target was shown during movement. In our tasks where multiple targets were presented, all targets were all valid choices and none of them disappeared. This difference has important repercussions on the model used to evaluate the decisions as (i) we do not have to consider the uncertainty about a target being cued into the decision processes and (ii) the alternative target can be selected even late in the movement if it suddenly becomes more desirable (in terms of cost-to-go) than the current one.

In fact, the case of multiple targets can be simulated without using continuous monitoring of the cost-to-go (CTG): indeed, by minimizing the sum of square distances to several targets, the controller spontaneously aims for the barycenter. Then, changing the location of one target during movement produces the expected rerouting. This method is able to generate the experimental results in the series of studies implying *go-before-you-know* paradigms (see simulations below) but is unable to simulate our results as all the targets should remain available throughout movement.

We addressed the reviewer concern by 1.) putting our present work in the context of the literature of go-before-you-know work (see **lines 468-477**) and 2.) demonstrating below that the behaviors observed in Chapman et al. 2010 and Wong et al. 2017 can be obtained without the need of a continuous tracking of the cost-to-go functions, which differentiates our current work from these previous studies. We also added a description of these additional simulations to the revised version of the manuscript (**lines 470-472**).

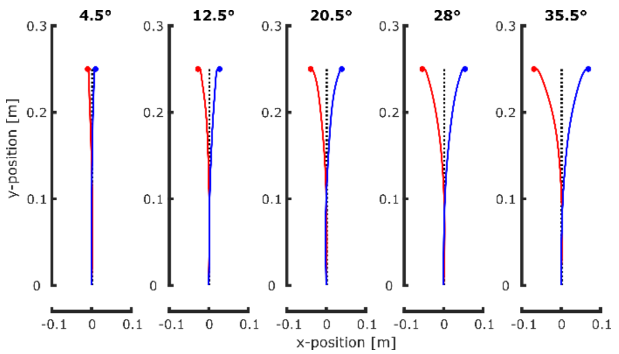

Figure 1 -Simulated results of the go-before-you-know paradigm in a condition similar to (Wong & Haith, 2017) in which the two potential targets are located on both sides of the original position. The correct target is cued after movement was initiated (after 10 timesteps in our model). The first portion of the movement reaches towards an average position of the two targets, whatever the distance between both targets. Different colors represent different cued targets.

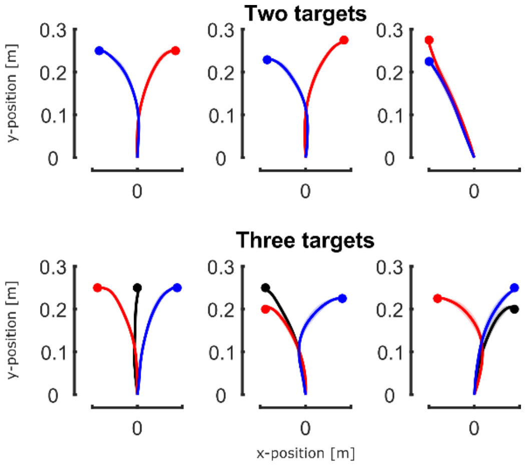

Figure 2 – Simulated results of the go-before-you-know paradigm in a condition similar to (Chapman et al., 2010) in which multiple potential targets are presented at different locations of the workspace. The correct target is cued after movement was initiated (after 10 timesteps in our model). The first portion of the movements reaches towards the barycenter of the different targets. Different colors represent different cued targets.

"By introducing the cost-to-go as a decision variable, we bridged an important gap between current models of motor control and decision making. We used the cost-to-go as a decision variable because of its theoretical grounding in the dynamic programming solution of the optimal control problem (54). It has been used to derive locally and globally optimal solutions to the control problem through stochastic optimal control (27,55) and reinforcement learning (56,57). The implementation presented in this paper allows to go beyond standard static formulation of OFC models with fixed parameters, and explicitly integrate the reward, changes in target structure and multiple targets in the cost-to-go, similarly to what was done with the value function (i.e. the opposite of the cost-to-go) in reinforcement learning (58). It is important to mention previous studies investigating how humans performed reaching movements in a paradigm where two or more targets were presented prior to movement, and the correct one was cued later (59–62). The behavior observed in this go-before-you-know paradigm can be reproduced by averaging the control policies or target locations associated with the different options (62–65), simulations not shown. Thus, from a computational perspective this problem does not require to update the controller online which differs from the experimental results that we modelled. In addition, in our task, no target was cued before or during movements, the controller must decide between different valid choices instead of waiting to see which one is correct. However, a controller endowed with dynamic updates based on continuous tracking of the cost-to-go can also handle the problem of reaching to uncertain targets."

Minor Concerns

1. Recent work in both reaching *(Mehrabi, N., Sharif Razavian, R., Ghannadi, B., & McPhee, J. (2017). Predictive simulation of reaching moving targets using nonlinear model predictive control. Frontiers in computational neuroscience, 10, 143; Bashford, L., Kobak, D., Diedrichsen, J., & Mehring, C. (2022). Motor skill learning decreases*

movement variability and increases planning horizon. Journal of Neurophysiology, 127(4), 995-1006.) and gait *(Darici, O., & Kuo, A. D. (2022) Humans plan for the near future to walk economically on uneven terrain. arXiv preprint arXiv:2207.11224.)* highlight the horizon of planning and or MPC. Can the authors expand in the introduction, with an additional paragraph, the works that have used MPC or considered the horizon of control to understand sensorimotor control in the literature.

We adjusted the introduction (**lines 96-97**) and discussion (**lines 434-435**) sections to mention the notion of control horizon. Even though the control horizon of movement (reaching, locomotion) is an important topic of research, we believe that this is beyond the scope of this paper. However, as the time horizon is also a cost parameter, it could be updated following the same approach as the one that we propose to account for updates in timing constraints that could occur.

"We modeled online modifications in control by implementing a receding horizon controller inspired by model predictive control (33,34), which was previously used to study sensorimotor control (35–37)."

"Thanks to the recursive computations of the feedback gains inherent to this framework, Model Predictive Control was also used to investigate the control horizon in sensorimotor control (35–37)."

2. Last paragraph of the discussion. Decision-making literature, much of the work pioneered by Paul Cisek, suggests that decision-making and control are intertwined. For the past two decades the general thought has been that the nervous system does not plan, decide, act. This would suggest that the cost-to-go is continually monitored, in terms of both energetics and evidence-based decision-making processes. Can the authors please comment and potentially adjust this paragraph.

We adjusted the last paragraph (**lines 508-509**) of the discussion to emphasize our hypothesis that the cost-to-go is continuously monitored during movement such that decisions can be made by considering energetic and performance factors as suggested in the decision-making theory of (Cisek, 2012). Our work provides modeling and experimental support for this theory in the control of reaching movement.

"Together our modeling results expand the applicability of optimal feedback control framework to tasks involving dynamic changes in parameters during movement and formalize the distributed consensus model suggested by Cisek (52) to the control of reaching movements."

3. In the decision-making and foraging literature would suggest the cost is reward rate = (reward - energy) / time (Carland, M. A., Thura, D., & Cisek, P. (2019). The urge to decide and act: implications for brain function and dysfunction. The Neuroscientist, 25(5), 491-511). Here rewards are added at a higher decision-making level. It is plausible that rewards themselves influence feedback gains. Can the authors please comment, and perhaps add to the paragraph currently in the discussion.

We are aware of the effect of reward on movement duration and specifically the notion of cost-of-time (Haith et al., 2012; Shadmehr et al., 2010) and we even observed in our previous work that reward increased feedback responses (De Comite et al., 2022b). We detailed in the revised version of the manuscript additional modulations of the controller that would be required to implement this notion in our model (**lines 495-500**). Such developments could for instance involve infinite time horizon of the controller (Qian et al., 2013; Rigoux & Guigon, 2012) as they implicitly allow for this flexibility. We believe that it is an important question for prospective work.

"Recently, the influence of uncertainties about the environment (85,86) and reward (6,21) on reaching behavior has been reported. This can be modeled thanks to the use of a robust controller (86). To account for the modulation of

movement velocities and feedback gains related to temporal factor or to the use of a robust strategy, a fuller implementation requires an additional module which not only selects the current target or controller, but also modifies the control gains according to the vigor or robustness of the intended movement."

4. A classic prediction from Fitt's law is that participants move faster when reaching to big targets than small targets. Do the previous data set find that wider targets led to faster movement? Are movement times constrained in these experiments? If so, what would happen with forward velocity if movement times were not constrained? In the paper the authors manipulate only the x-dimension of the cost function to manipulate target size by manipulating the position state cost. I assume that this does not lead the model to predict faster forward velocity when reaching towards larger targets. Can the authors please comment and include in the discussion.

In all the experiments simulated here, the movement duration was fixed a priori as customary with the finite horizon formulation of optimal feedback control (Todorov & Jordan, 2002). Further model developments, interacting with the movement horizon are required to fully answer this question and we believe that it is an interesting challenge for prospective work. This point was added to the paragraph of the discussion covering the limitations of our model (**lines 501-506**). We also clarified our modeling choice for the movement duration in the methods section (**lines 607-608**).

"Another important aspect that our model does not cover is the modulation of movement duration with various parameters such as reward (5,69,81,87) or required accuracy (88). We chose to use a finite horizon controller in our different simulations since we believe that it would not affect our conclusions. Modulations of movement duration cannot naturally emerge from our model and the incorporation of updates in movement times requires further developments either by considering a receding horizon (84) or an infinite horizon (89,90)."

"The time horizon (N) was decremented at each time step such that the total movement time remained constant"

5. Paragraph 442-452. The ideas of the influence on movement speed due to reward or due to environmental uncertainty are shown to influence forward velocity. But these two are not the same underlying mechanism. The real question is how reward influences the feedback gains, and how environmental uncertainty influences feedback gains, which are likely very different mechanisms. Also, I know the authors have used the word vigor here to discuss feedback gains / forward velocity in the past. However, in general, vigor is more colloquially known in the field as a product of reward.

This is a fair point, and we added some nuances in the paragraph of the discussion covering that point. More specifically, we aligned our definition of vigor to the one colloquially known in the field and adjusted the paragraph to clarify this aspect (**lines 495-500**).

"Recently, the influence of uncertainties about the environment (85,86) and reward (6,21) on reaching behavior has been reported. This can be modeled thanks to the use of a robust controller (86). To account for the modulation of movement velocities and feedback gains related to temporal factor or to the use of a robust strategy, a fuller implementation requires an additional module which not only selects the current target or controller, but also modifies the control gains according to the vigor or robustness of the intended movement."

6. line 105-108: Can the authors expand on how decision-times predicted from their model would differ from diffusion models.

We expect that decision times resulting from drift-diffusion models should be similar to the ones we presented here even though we acknowledge that more targeted modelling work should be conducted to answer these questions. Specifically, we suggest that a generalization of the drift-diffusion model for motor decisions can be obtained by using the cost-to-go as decision variable, and the difference of cost-to-go across targets as decision boundary, such that movement is rerouted when an alternative target becomes more attractive. We clarified the part of the introduction referring to these models to avoid any confusion (**lines 107-110**).

“Indeed, these models assume that decisions occur through the accumulation of a decision variable until it reaches a decision threshold. In the case of online control, we suggest that the decision variable is the cost-to-go and the threshold for the decision can be defined based on the difference of cost-to-go between alternative targets.”

7. line 198. Use another phrase than ‘thanks to’.

We replaced “…thanks to the motor commands” by “… using the motor commands…” (**line 216**).

“We also captured the patterns of activations in the stretched muscles reported in the experimental study using the motor commands along the x-axis.”

Reviewer 2

This article presents a model which accounts for human motor behavior following perturbations. It is based on optimal feedback control combined with monitoring of the expected cost-to-go function to decide about motor goal. The model explains a broad range of observations collected in previous experiments from the authors and from other research groups.
The article is well written and rather easy to read. The simulations are properly run and the results are convincing.
I have four comments:

1. line 116 "We extended the optimal feedback control framework ...", line 544 "we propose a recursive dynamical computation ...". I am perplex about these statements. In fact, the OFC framework intrinsically embeds these characteristics (Bryson and Ho 1975, Applied Optimal Control; Todorov and Jordan 2002, Nat Neurosci 5:1226). Even if the control low can be precalculated (Eq. 8), it is implicit in the view of Todorov and Jordan that it is recalculated at each timestep to take into account changes in the body and the environment. There is no need to invoke a recursive dynamical computation nor MPC, in particular because MPC involves a receding horizon (moving horizon; fig. 1 in Garcia et al. 1989, Automatica 25:335), which is not the case here (see below). In the same way, monitoring the cost-to-go function is not a novelty (Todorov and Jordan 2002; Todorov 2004, Nat Neurosci 7:907) and has been used for decision making in the framework of motor control (Rigoux and Guigon 2012, PLoS Comput Biol 8:e1002716). I would suggest to clarify these issues.

We thank you for this comment and we understand that the related aspects needed to be clarified. We think that the original formulation of the optimal feedback control model proposed by Todorov (Todorov & Jordan, 2002) did not implicitly mention that the feedback gains were recalculated during movement. Changes in the body

and environment (e.g. mechanical perturbations or target jumps), that generate a change in the state vector do not require an explicit recalculation of the control policy. On the contrary, fundamental changes in the task demands such as the target structure or the decision to switch to another target require this re-computation because they modify the control gains. We clarified this point in the revised version of the manuscript on **lines 86-87** and **lines 415-417** by specifying that our implementation consists of a re-computation of the feedback gains which leverages a continuous monitoring of the cost-to-go.

"Even though the OFC framework has been extensively used to explain flexible feedback control (2,29–32), it was mainly applied to static environments: that is, cost parameters have been typically considered fixed during a given trial. Therefore, a mechanism able to respond to these sudden changes in task parameters as observed in previous experimental studies is required."

"The addition of this decision module to the native OFC implementation is necessary as it is often assumed that the cost function, and therefore the feedback gains, are fixed during movement, which was not compatible with the experimental results captured in this study."

We agree with the reviewer that our model is not strictly speaking MPC since we did not implement a receding horizon (which is why we mentioned that it was a variant of MPC with non-fixed horizon). A direct extension of our model could be to feature updates in movement time following the model of Rigoux and Guigon (Rigoux & Guigon, 2012). To avoid any confusion on that point, we clarified the introduction (**line 96**), discussion (**lines 436-437**) and methods (**lines 607-608**) sections to emphasize this important distinction and pointed to additional references to contextualize our work.

"We modeled online modifications in control by implementing a receding horizon controller inspired by model predictive control (33,34), which was previously used to study sensorimotor control (35–37)."

"Here, we took inspiration from this MPC framework to combine the recomputation of the control policy with the continuous evaluation of the cost-to-go function through a generalization of a previous…"

"The time horizon (N) was decremented at each time step such that the total movement time remained constant."

2. The model involves a 50-ms delay for sensory feedback and a 150-ms delay to update the control policy. The latter delay seems rather adhoc. It could be more conservative to say that there is a single feedback delay corresponding to the time it takes to detect changes in the body or the environment from sensory information. The idea that it takes time to update the control policy is related to the use of the LQG framework and the fact that the control policy is task-dependent (for a different view, see Guigon 2023, Psychol Rev 130:23).

We must clarify that these delays are constrained by the physiology and by empirical observations. The 150ms delay used to update the control policy was determined based on previous experimental work (De Comite et al., 2021), where we observed that participants updated their controller within this amount of time when the target structure was changed during movement. The delay of 50ms corresponds to the long-latency delay in the human nervous system, which function is similar to a state-feedback control (Crevecoeur & Kurtzer, 2018; Pruszynski & Scott, 2012). We clarified this point in the manuscript in the results (**lines 147-148**) and methods (**lines 642-644**) sections.

"We simulated this experiment and used the 150ms delay as a parameter to update the control policy based on the actual target structure, which differed from the 50ms delay in the feedback loop associated with physiological delays of long-latency responses (39,40)."

"Importantly, this delay is different from the one associated with the long-latency feedback responses elicited by mechanical perturbations (39,40) as it requires to perform an additional update of the control policy (17,49)."

3. line 98 "We modeled online modifications in control by implementing a receding horizon controller inspired by model predictive control", line 549 "Q(t) and N are adapted to respectively capture time-varying task demands and changes in movement horizon". I see nowhere in the article what is the value of the receding horizon, and whether and how N is actually updated. This is a critical issue for online motor decision between alternative goals. Cost calculation is dependent on intended movement duration. In a normative approach (e.g. Shadmehr et al. 2010, J Neurosci 30:10507; Rigoux and Guigon 2012), a rewarded goal can become more or less interesting depending on the intended time to reach the goal (Stevens et al. 2005, Curr Biol 15:1855). The proposed model seems to bypass this issue.

We agree with the Reviewer that, strictly speaking, we did not implement a receding horizon controller as the movement duration was fixed in all the simulations we ran. Our model can however be extended to receding horizon, possibly of varying duration dependent on rewards and the cost of time. We adjusted the revised version of the manuscript as described above to remove any ambiguity regarding the time horizon of our model (**lines 607-608**) and mention the possible extension of our model to receding horizon formulations (**lines 495-502**).

"The time horizon (N) was decremented at each time step such that the total movement time remained constant."

"Recently, the influence of uncertainties about the environment (85,86) and reward (6,21) on reaching behavior has been reported. This can be modeled thanks to the use of a robust controller (86). To account for the modulation of movement velocities and feedback gains related to temporal factor or to the use of a robust strategy, a fuller implementation requires an additional module which not only selects the current target or controller, but also modifies the control gains according to the vigor or robustness of the intended movement. Another important aspect that our model does not cover is the modulation of movement duration with various parameters such as reward (5,69,81,87) or required accuracy (88)."

4. As a minor point, I would suggest, for each simulation result, to add a pointer to corresponding figures in the corresponding experimental articles. For instance, the simulation results in Fig. 1 are related to experimental results in De Comite et al. (2021).

This was added to the revised version of the manuscript, see the figure captions.

Reviewer #3

This paper by Comite, Lefevre, and Crevecoeur proposed a switching mechanism of the cost-to-go during the optimal feedback control problem in humans and animals. During goal-directed reach movements tasks where the requested movement is slow and not ballistic, this proposed architecture is able to switch the optimal control policy in response to the change of the task goal. This model replicated the number of previously reported behavioral results. The proposed hypothesis is modestly new, and the proposed formulation of the model is solid. However, I have several concerns that I would like the authors to address before this gets published. Especially, the method section has to be more detailed and additional simulation studies to explain the characteristics of this model are necessary.

Major concerns

1) Since OFC based model is very flexible in that the setting of the cost function determines its generated behavior, it can explain any behavioral characteristics by manually tuning the cost function for the simulation. In other words, the computational model based on OFC often lacks falsifiability. Thus, setting an alternative hypothesis and refuting this alternative possibility is crucial for a scientific paper. In this paper, the author explains the simulation results for explaining the previous reports straightforwardly without setting an alternative hypothesis explicitly. One alternative hypothesis they discussed is the target switching hypothesis, which predicts the fixed response time.

(although I think this prediction is too specific, which is not always true for the switching hypothesis). For example, we can think about another possibility, such as the continuous update of the target representation in the brain is modulated by the reward information. This hypothesis also seems to be derived from previous studies since continuous target remapping based on Bayesian integration has been proposed and discussed in the motor learning literature (e.g., S Vaziri JNS 2006). My intention here is not to propose a specific alternative hypothesis. Rather, I recommend that the authors consider several potential hypotheses from the previous literature and then formalize them as computational models. Model comparisons of multiple models of alternative hypotheses make this paper much stronger.

We thank the reviewer for this important remark. The central contribution of our paper is to formalize, within the OFC framework, the existence of within-movements updates of the controller structure that could explain the rapid changes in behavior observed experimentally. We need an alternative hypothesis that is able to falsify our interpretation of the data, but not the OFC model itself. Since we hypothesized that the cost-to-go (CTG) is used during movement to adjust the control policy, a clear alternative is that it is not used online (H0). In such case, we should not observe any update in control and any switch in reached target dependent on the target rewards and perturbations, which is inconsistent with the experimental findings (Cos et al., 2021; De Comite et al., 2021; Martí-Marca et al., 2020). Therefore, H0 is falsifiable, and the empirical data is sufficient to reject it.

Then, we specifically proposed that this tracking of the cost-to-go occurs continuously. The alternative hypothesis would be that it is event-triggered (e.g. occurred only after perturbations). In this case, we should not observe any difference in decision times for different reward distributions or perturbation magnitudes, which we find in our data. Again, our interpretation was falsifiable but the data allowed us to reject our alternative hypotheses.

In all, we emphasized that our claims were falsifiable but that they were supported by empirical observation. We added throughout the revised version of the manuscript clearer descriptions of these different alternative hypotheses and how they were inconsistent with the experimental data (**lines 153-159**, **219-222**, **293-296**, and **340-345**).

"An alternative hypothesis would be that, in the absence of a module dedicated to the detection of the alternative options and to the alteration of the controller structure, the expected behavior should correspond to the one observed when reaching for the initial target (narrow square and wide rectangle for the narrow-to-wide and wide-to-narrow conditions respectively). Under this assumption, we would observe a larger end-point error for the wide-to-narrow conditions (full line blue, Figure 2A) and a larger corrective response for the narrow-to-wide condition (full line red, Figure 2E), which is inconsistent with the experimental data (17)."

"The fact that different changes in target structure have different impact on behavior, rules out the alternative hypothesis of an ad hoc adjustment of the controller that would be determined before movement started and independent of the rate of change in target structure."

"Since we did not impose the goal target at any time during movement we observed, for a same experimental condition (reward distribution and force amplitude), different behaviors (see Figure 4A, different colors for a same panel). This flexibility to decide could not be reproduced if the controller only considered a single reach target for the current movement."

"Our interpretation is falsifiable in two ways: a first alternative hypothesis could be that the cost-to-go is not recalculated online following changes in task demands and that there is no module able to adjust the control policy online. This first hypothesis could be rejected based on the experimental observations described above. The second alternative hypothesis would be that the evaluation of the cost-to-go that underlies motor decisions is not continuous but instead is triggered by discrete events such as perturbations or visual cues."

2) The most important new concept in the proposed computational model has not been well described in the main

section. For instance, Figure 6, schematic diagrams of this model, was referred to in the method. To make the scientific argument clear, this should appear in Figure 1 (main or intro) instead of Figure 6 (method)

We added a new figure at the beginning of the paper to emphasize the novelty of the concept proposed in the computation model (see Figure 1 in the revised manuscript).

3) L 407, "the cost-to-go is monitored continuously" The word monitor is not appropriate here since the selection mechanism of the different cost-to-go was not proposed and not examined. A possible mechanism is, for example, competition among different cost-to-g. In this case, nothing monitors cost-to-go, and one option is automatically and spontaneously selected as a result of the competition. Instead, "the hypothesis that the different cost-to-go are computed continuously" is appropriate.

We apologize for our confusing formulation and revised it through the manuscript to clarify the fact that the cost-to-go of the different alternatives are compared and, whichever motor command is associated with the lower value will be applied to the system. This was modified throughout the manuscript (**see lines 445-447** and the manuscript **title** for instance).

"Our main contribution is to provide support for the hypothesis that the cost-to-go of the different alternatives are continuously compared and the motor command associated with the lowest value is selected, which…"

4) The model (Eqn(4)) does not have signal dependent nose. The signal-dependent noise and the smooth minimum variance trajectory is the most important clue of the OFC theory. These SDN should largely influence the simulated behavior. I recommend the author incorporate SDN into the model. If the proposed model for predictive cost-to-go switching architecture can't accommodate SDN, the model should not be plausible.

We indeed choose to present our model without signal dependent noise in the paper for simplicity. However, as suggested we reproduced below some of our experimental results with signal dependent noise (implemented as developed in (Todorov, 2005)) to verify that our conclusions were amenable to this known feature of the human sensorimotor system (see figure below). The presence of signal-dependent noise affected the expression of the cost-to-go function but did not alter the impact of rewards and target structure that we introduced in the present study (see equation (11) in (Crevecoeur et al., 2011), the last term of the cost-to-go is similar to ours). In the revised version of the manuscript, we mention that our results are robust to the addition of signal-dependent noise (see **lines 688-693**).

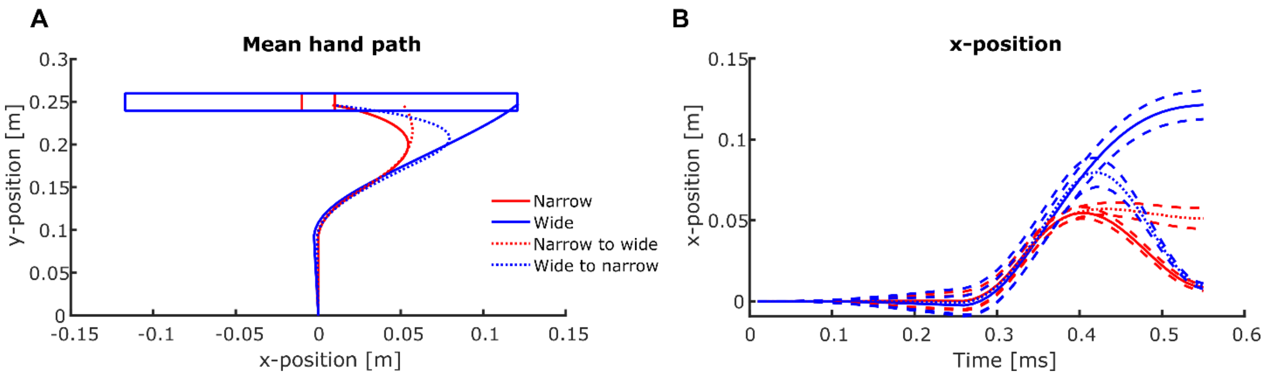

*Figure 3 – Simulations of the first task presented in the manuscript with signal dependent noise added to the state-space equation update and to the feedback equation. **A** Mean hand path in the different conditions in presence of a rightward mechanical perturbation. The dotted lines represent the trials in which the target structure changed during movement. **B** Mean*

and standard deviation of the Lateral hand deviation during movement for the different target conditions in presence of signal-dependent noise (SDN). The dotted lines represent the trials during which the target structure changed during movement.

"The system dynamics we considered in the present study does not involve signal-dependent noise for clarity (see Equation (4)), but we verified that this simplification did not affect the modeling results as it impacts the cost-to-go function independently of the modification we suggested (see Equation 11 in (96)). We therefore decided to only consider additive noise in the present work, simulations for the signal-dependent noise not shown."

5) Most of the idea of the proposed computational model has already been proposed in Nashed(25). Although there is an original contribution of this present paper, such as the offset of the cost-to-go by reward, the main argument of this paper (online switch of the cost-to-go) has been proposed in Nashed(25). Then, which is the real novelty of the present paper? If the reward bias is the original contribution, the introduction has to be rewritten.

It is clear that our work builds on this and other previous studies, and we agree that the novelty of the current paper needed to be better articulated. The work proposed by Nashed et al. (Nashed et al., 2014) used the idea of continuous monitoring with targets of equivalent rewards as a candidate mechanism but without empirical support to the specific idea of continuous calculation of the cost-to-go. The novelty of the current work is to (1) extend this idea to multiple target rewards, structures, as well as dynamic changes in target shape, and (2) to highlight the different decision times dependent on rewards and actual trajectories, compatible with the continuous calculation of the cost-to-go. We therefore started from the same idea but were able to explain a broader class of phenomena and to provide empirical evidence for our hypotheses. The novelty of our work was clarified in the introduction (**lines 86-87**) and discussion (**lines 415-417**) sections of the revised manuscript.

"Even though the OFC framework has been extensively used to explain flexible feedback control (2,29–32), it was mainly applied to static environments: that is, cost parameters have been typically considered fixed during a given trial. Therefore, a mechanism able to respond to these sudden changes in task parameters as observed in previous experimental studies is required."

"The addition of this decision module to the native OFC implementation is necessary as it is often assumed that the cost function, and therefore the feedback gains, are fixed during movement, which was not compatible with the experimental results captured in this study."

6) How exactly the endpoint reward offset (say, r_b(N)) biases the s_t is unclear. This part is not well written in the method section (l638-l642). Considering the algorithm of the OFC solution, the backward computation of DP (Eqn 16), taking account of this bias, should be needed. A detailed additional explanation using equations is necessary.

The reward bias only affects the cost-to-go function by offsetting it. It has no impact on the feedback gains and on the control policy. We updated the methods section of the manuscript to clarify this point and explicitly indicate how this manipulation affects the different terms, **see lines 713-721**.

7) The equations of the selection policy of the cost-to-go v_i,v_j (L647) should be needed. How does the action selection probability is computed? Was the soft-max-function was used? Then, please discuss the reason why this is rational.

This is an important point which was also highlighted by Reviewer 1. The experimental paradigms we studied differed from the *go-before-you-know* ones in which the correct target is cued after movement onset. In our case, no target was cued after movement onset and the controller succeeds the task if it reaches any of these. Action selection is therefore based on a maximum function rather than on a soft max function, which could for

instance map to different targets having different probability of being cues instance, the different targets had different probability of being cued. The decision process that we implemented is a winner-takes-all where, at any time the action associated with the lowest cost-to-go is selected. We clarified that important difference in the discussion (**lines 468-477**) and methods (**line 726**) sections of the revised version of the manuscript.

"It is important to mention previous studies investigating how humans performed reaching movements in a paradigm where two or more targets were presented prior to movement, and the correct one was cued later (59–62). The behavior observed in this go-before-you-know paradigm can be reproduced by averaging the control policies or target locations associated with the different options (62–65), simulations not shown. Thus, from a computational perspective this problem does not require to update the controller online which differs from the experimental results that we modelled. In addition, in our task, no target was cued before or during movements, the controller must decide between different valid choices instead of waiting to see which one is correct. However, a controller endowed with dynamic updates based on continuous tracking of the cost-to-go can also handle the problem of reaching to uncertain targets."

"Then, at each time step, the cost-to-go functions v^i associated with each option i are computed and compared, including rewards that were not related to the ongoing movement throughout the terms s_t^i, and the motor command associated with the smallest cost-to-go function amongst all the options is selected."

8) There is a gap in the logic between the conventional OFC model (Eqn 15-18) and the RL model-like value estimate in Eqn(19). Please explain more about the reason why the formulation of Eqn(19) was necessary.

We acknowledge the presence of the gap in the previous version. The expression of the cost-to-go function, assuming an optimal policy can be formally proved by using Dynamic Programming and Bellman equation. We provided a more detailed explanation about these steps and provided references to the proof in the case of reaching movement. We also highlighted the similarities between the cost-to-go function in the optimal control problem and the value function in the reinforcement learning problem, see lines **683-688**.

9) L264. I did not understand how the perturbation was incorporated into the OFC and how this perturbation altered the cost-to-go. This part should be clearly explained in the method section.

The perturbations were simulated by changing the value of the variable F_{ext} (**see lines 529-534**) in the state vector. The perturbation influenced the cost-to-go indirectly through its impact on the limb trajectory. We added this explicitly in the revised version of the manuscript (**lines 538-541**).

"To simulate the presence of a mechanical perturbation to the system we changed the value of F_{ext} to the amplitude of the perturbation. This perturbation amplitude will propagate to the subsequent timesteps thanks to Equation 3."

10) L318. The reader would like to understand the reason why RT becomes different in this model. What is the factor that alters RT? Is the estimated hand position matter, the variance of the estimate matter, or both matter? Additional simulations study (sensitivity analysis) is needed here to show the sensitivity of independent variables to the RT. The current explanation is not done well step-by-step.

The differences in response time observed in the decision task are produced because the cost-to-go functions are dependent on the target reward and hand trajectory. For a given distribution of reward, each target has its own basin of attraction in the state-space which corresponds to the set of states for which this target has the lowest cost-to-go. When the perturbation occurs, different perturbation amplitudes will yield different

kinematics which will result in longer or shorter times until the region of states attracted to the alternative target is reached.

We manipulated the main underlying factors (i.e. reward distribution and perturbation amplitude) both experimentally and in the simulations to characterize this modulation of decision time (see Figure 6 in the revised manuscript). The modulations observed in the simulations matched the ones that were reported experimentally.

To further answer the Reviewer's comment, we performed a sensitivity analysis to characterize the impact of additive motor and sensory noise on the decision time: we reproduced the results from Figure 4A of the manuscript with different values of motor and sensory noise (amplified up to 100 times the value of the manuscript results to remains in reasonable range of values). We quantified the modulation of the decision time as a function of both the noise amplitude and the variability of the trajectory at perturbation onset. We observed that for all levels of noise and variability, the decision time for the condition where the central target was more rewarding (red in the Figure 4 below) were longer than the conditions where all the targets had the same reward (black in the Figure 4 below). An increase in motor noise increases the decision time (Figure 4A) while an increase in sensory noise does not significantly modulates it (Figure 4B). We observed that the decision time seems to plateau for high values of motor noise (Figure 4 A) and is independent on the sensory noise. The difference in decision time between both conditions (same and different) are preserved across noise level even if it tends to be reduced for high values of motor noise. We mentioned it in the revised version of the manuscript (**lines 372-375**).

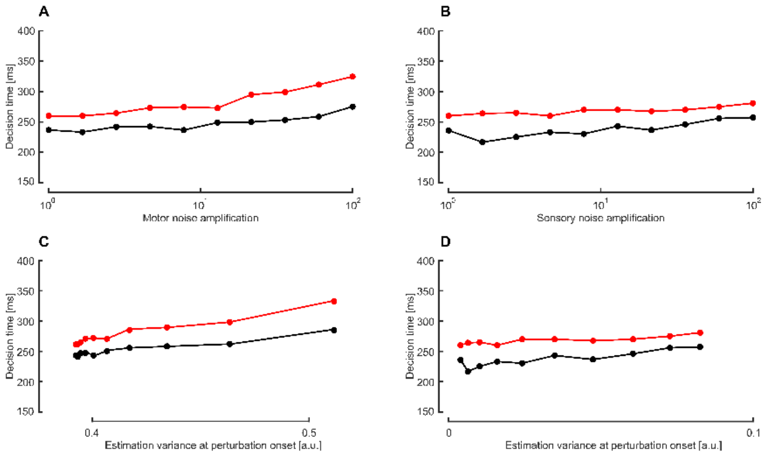

*Figure 4- **A** Modulation of the decision time (in ms) as a function of the motor noise amplification (relative to the one presented in the paper) in the conditions where all the targets had the same reward (black) and the condition where the central target had more reward (red). **B** Modulation of the decision time (in ms) as a function of the sensory noise amplification (relative to the one presented in the paper) in the condition where all the targets had the same reward (black) and the condition where the central target had a larger reward (red). **C** Modulation of the decision time as a function of the variance of the state estimation at perturbation onset for varying amount of motor noise, corresponding to the amplification values of panel **A**. **D** Modulation of the decision time as a function of the variance of the state estimation at perturbation onset for varying amount of sensory noise, corresponding to the amplification values of panel **B**.*

"This decision time is also impacted by the amount of motor noise (simulations not shown), which increases the decision time at higher level of noise but does not affect the difference between both reward conditions"

Minor

1) Please add the plots of the original behavior data, adapting it from the original paper. Otherwise, it is inconvenient to understand the simulation results.

We understand the Reviewer's comment but want to emphasize that our model, in line with other simulations of reaching movements, provides a qualitative framework to study the control of movements. Since the model approximates or simplifies certain characteristics of the biomechanical model, direct quantitative comparisons will be misleading. We therefore chose to not directly include the empirical data in the figures but added in each of the captions a pointer to the associated figures in the different papers so that the readers can compare these results.

2) L561, Eqn 11, "*" of "100*" is confusing and not accurate as a mathematical notation. Please delete them.

We modified that part of the methods section to integrate the Reviewer's comment (**lines 621-622**).

*"...where the * superscript refers to the target state, $p(t)$ is the position at time t, the subscripts refer to the x- and y-coordinates, w_1 and $w_2 = 0$ are the weights associated with the narrow and wide dimensions of the targets."*

Chapman, C. S., Gallivan, J. P., Wood, D. K., Milne, J. L., Culham, J. C., & Goodale, M. A. (2010). Reaching for the unknown : Multiple target encoding and real-time decision-making in a rapid reach task. *Cognition*, *116*(2), 168-176. https://doi.org/10.1016/j.cognition.2010.04.008

Cisek, P. (2012). Making decisions through a distributed consensus. *Current Opinion in Neurobiology*, *22*(6), 927-936. https://doi.org/10.1016/j.conb.2012.05.007

Cos, I., Pezzulo, G., & Cisek, P. (2021). Changes of mind after movement onset : A motor-state dependent decision-making process. *eNeuro*, *8*(6), ENEURO.0174.

Crevecoeur, F., & Kurtzer, I. (2018). Long-latency reflexes for inter-effector coordination reflect a continuous state feedback controller. *Journal of Neurophysiology*, *120*(5), 2466-2483. https://doi.org/10.1152/jn.00205.2018

Crevecoeur, F., Sepulchre, R. J., Thonnard, J. L., & Lefèvre, P. (2011). Improving the state estimation for optimal control of stochastic processes subject to multiplicative noise. *Automatica*, *47*(3), 591-596.

Crevecoeur, F., Thonnard, J.-L., & Lefevre, P. (2020). A very fast time scale of human motor adaptation : Within movements adjustments of internal representations during reaching. *eNeuro*, *7*(1).

De Comite, A., Crevecoeur, F., & Lefèvre, P. (2021). Online modification of goal-directed control in human reaching movements. *Journal of Neurophysiology*, *125*(5), 1883-1898.

De Comite, A., Crevecoeur, F., & Lefèvre, P. (2022a). Continuous Tracking of Task Parameters Tunes Reaching Control Online. *ENeuro*, *9*(4). https://doi.org/10.1523/ENEURO.0055-22.2022

De Comite, A., Crevecoeur, F., & Lefèvre, P. (2022b). Reward-Dependent Selection of Feedback Gains Impacts Rapid Motor Decisions. *Eneuro*, *9*(2), ENEURO.0439-21.2022. https://doi.org/10.1523/ENEURO.0439-21.2022

Haith, A. M., Reppert, T. R., & Shadmehr, R. (2012). *Evidence for Hyperbolic Temporal Discounting of Reward in Control of Movements*. *32*(34), 11727-11736. https://doi.org/10.1523/JNEUROSCI.0424-12.2012

Ikegami, T., Ganesh, G., Gibo, T. L., Yoshioka, T., Osu, R., & Kawato, M. (2021). Hierarchical motor adaptations negotiate failures during force field learning. *PLOS Computational Biology*, *17*(4), e1008481. https://doi.org/10.1371/journal.pcbi.1008481

Knill, D. C., Bondada, A., & Chhabra, M. (2011). Flexible , Task-Dependent Use of Sensory Feedback to Control Hand Movements. *Journal of Neuroscience*, *31*(4), 1219-1237. https://doi.org/10.1523/JNEUROSCI.3522-09.2011

Martí-Marca, A., Deco, G., & Cos, I. (2020). Visual-reward driven changes of movement during action execution. *Scientific Reports*, *10*(1), 1-12. https://doi.org/10.1101/656330

Nashed, J. Y., Crevecoeur, F., & Scott, S. H. (2012). Influence of the behavioral goal and environmental obstacles on rapid feedback responses. *Journal of Neurophysiology*, *108*(4), 999-1009. https://doi.org/10.1152/jn.01089.2011

Nashed, J. Y., Crevecoeur, F., & Scott, S. H. (2014). Rapid Online Selection between Multiple Motor Plans. *Journal of Neuroscience*, *34*(5), 1769-1780. https://doi.org/10.1523/JNEUROSCI.3063-13.2014

Pruszynski, J. A., & Scott, S. H. (2012). Optimal feedback control and the long-latency stretch reflex. *Experimental Brain Research*, *218*(3), 341-359.

Qian, N., Jiang, Y., Jiang, Z.-P., & Mazzoni, P. (2013). Movement Duration, Fitts's Law, and an Infinite-Horizon Optimal Feedback Control Model for Biological Motor Systems. *Neural Computation*, *25*(3), 697-724. https://doi.org/10.1162/NECO_a_00410

Rigoux, L., & Guigon, E. (2012). A Model of Reward- and Effort-Based Optimal Decision Making and Motor Control. *PLoS Computational Biology*, *8*(10), e1002716. https://doi.org/10.1371/journal.pcbi.1002716

Shadmehr, R., Orban de Xivry, J.-J., Xu-Wilson, M., & Shih, T. (2010). Temporal Discounting of Reward and the Cost of Time in Motor Control. *Journal of Neuroscience*, *30*(31), 10507-10516. https://doi.org/10.1523/JNEUROSCI.1343-10.2010

Todorov, E. (2005). Stochastic Optimal Control and Estimation Methods Adapted to the Noise Characteristics of the Sensorimotor System. *Neural Computation*, *17*(5), 1084-1108. https://doi.org/10.1162/0899766053491887

Todorov, E., & Jordan, M. I. (2002). Optimal feedback control as a theory of motor coordination. *Nature Neuroscience*, *5*(11), 1226-1235. https://doi.org/10.1038/nn963

Wong, A. L., & Haith, A. M. (2017). Motor planning flexibly optimizes performance under uncertainty about task goals. *Nature Communications*, *8*, 1-10. https://doi.org/10.1038/ncomms14624
