# Supplementary material for: Continuous evaluation of cost-to-go for flexible reaching control and online decisions
Source: PLoS Comput Biol. 2023 Sep 27;19(9):e1011493. doi: 10.1371/journal.pcbi.1011493 (PMC10561875; doi:10.1371/journal.pcbi.1011493)
Supplement: Attachment — Submitted filename: ReviewersResponse.docx [file pcbi.1011493.s003.docx]

We would like to thank the Reviewers and the Editors for their in-depth assessment of our work and for raising the important points below. These helped us to significantly improve the paper. Please find below a point-to-point response to the comments that were made along with the references to the modifications of the text as well as snippets of the modified manuscript when relevant.

As with all papers reviewed by the journal, your manuscript was reviewed by members of the editorial board and by several independent reviewers. In light of the reviews (below this email), we would like to invite the resubmission of a significantly-revised version that takes into account the reviewers' comments.

Although the reviewers were mostly satisfied with the responses to the previous review comments, there remain a number of issues to be resolved. Specifically, the reviewers still had concerns about 1) The exact formulation of the model (receding horizon and model-predictive control?)  2) The need to reformulate details of the model to simulate different tasks in an ad-hoc way, and 3) How the findings might be impacted by signal-dependent noise.

Of these I believe that 1) still needs to be resolved. I agree with Reviewer 2 that the presentation of the model is unclear and I am similarly confused about how any aspect of the model relates to model-predictive control or a receding horizon. Is it just the idea that the optimal policy (or policies) are recomputed at each timestep? Line 95-97 introduce the model in terms of model-predictive control and receding-horizon control, but this seems to mismatches with the description of the implementation details.

Regarding points 2) and 3), I think these are important caveats to the current contribution, and should be clearly stated as such in the paper, but I don't view them as critical weaknesses. Ad-hoc tuning of models is a general issue with optimal control models, but this is fairly orthogonal to the main idea conveyed in the work (i.e. that the decision to switch between targets varies in a stereotyped way and this can be accounted for by the model). Similarly, while signal-dependent noise does provide a strictly more realistic model of the sensorimotor system, and there would certainly be additional challenges in extending these ideas to a signal-dependent noise setting (as Reviewer 3 highlights), the core conceptual idea can still be applied and I don't think the core insights from the LQG setting are invalidated.

Therefore, in your revision, please address the issues surrounding the confusion with model presentation, and ensure that the discussion adequately addresses the limitations and possible future extensions of the approach.

We addressed the concerns mentioned above and hope they will help remove the confusion with the model presentation and more directly state the limitations of our model. We reworked the Discussion section of the manuscript to emphasize the limitations of the model. More specifically, we explicitly mentioned that we needed to reformulate the cost function for each experiment as a consequence of the property of OFC that the control policy depends on the cost function (thus a change in policy implies a change in cost-function). We clarified the ambiguity concerning the time horizon of the model: movement duration is defined a priori and kept constant throughout the entire stimulations. Finally, we addressed the concern concerning the absence of signal-dependent noise by (1) adding an appendix which briefly explains how our model generalizes to a dynamical system with signal-dependent noise on the state and feedback dynamics, and (2) generating a supplementary figure which reproduce the most important results in presence of signal-dependent noise.

**Reviewer 1**

The authors have made several changes that have improved the manuscript. I just have a couple remaining comments. Please see latest comments below that are denoted, "LATEST COMMENT"

Specific concerns
1. This first point is probably the most important to address in the paper. Since its introduction to the field, optimal feedback control has been one of the most powerful frameworks to understand human behavior. What has been powerful about this approach is that a lot of behaviour emerges from the model. Here, however, the authors manipulate the cost function differently for each new experimental condition in an ad hoc fashion---rather than all the behaviour emerging from the same model, with parameters and cost function held constant, given task demands. In other words, applying a new cost function for each experimental condition in an ad hoc manner is less powerful than having behavior emerge from the same model. Can the authors please include this as a limitation within the discussion.

*We understand the Reviewer’s concern and discuss that point in more detail in the revised version of the manuscript. A single parametrization of the optimal feedback control model can indeed result in different behavioral realizations. Reaching behaviors in presence of perturbations (visual or mechanical) and the selective corrections for the effect of noise can be generated using the same cost-function as the one used to generate unperturbed movements (Todorov & Jordan, 2002). However, alterations of the target structure (Knill et al., 2011; Nashed et al., 2012), adaptation mechanisms to velocity-dependent force fields (Crevecoeur et al., 2020; Ikegami et al., 2021) or multiple potential targets (Nashed et al., 2014) necessitate adjustments of the controller, either by changing the cost-function to accommodate specific task requirement, or by changing the model parameters to represent adaptation. The set of tasks that we considered in the present study fall in the category where sudden changes of goal or target structure requires considering the respective cost-function of each configuration or option. The specific contribution of this paper is to demonstrate that the within-movement corrections observed experimentally can be predicted by updating the control policy online thanks to a continuous calculation of the cost-to-go. We clarified this point in the introduction (lines 86-87) and discussion sections (lines 415-417).*

*“Even though the OFC framework has been extensively used to explain flexible feedback control (2,29–32), it was mainly applied to static environments: that is, cost parameters have been typically considered fixed during a given trial. Therefore, a mechanism able to respond to these sudden changes in task parameters as observed in previous experimental studies is required.”*

*“To accommodate the experimental observations made about the dependency of these changes, we proposed an extended implementation of the OFC framework combined with a decision module based on a continuous observation of the target structure and an estimation of the cost-to-go function. The addition of this decision module to the native OFC implementation is necessary as it is often assumed that the cost function, and therefore the feedback gains, are fixed during movement, which was not compatible with the experimental results captured in this study.”*

LATEST COMMENT: The responses above, while helpful, I do not believe fully address this concern---which is also the related to the first major concern by reviewer 3 (and while the response there is also helpful, I would argue that this concern is not fully addressed and still needs to be mentioned as a limitation). Specifically, the theory / model becomes unfalsifiable when the cost function (independent of task dynamics) is changed for each unique experimental condition, which the authors have done here. Here the authors must hand tune the cost function for each experimental condition (e.g., dot vs bar, slow vs. fast, etc.). That is, there are as many degrees of freedom (by hand tuning) as there are experimental conditions, so it is not possible to falsify. An alternative would be that between / within trial adaptation or perhaps some other time-dependent process naturally changes the cost function, but that is not done in this paper.

Conversely, a model is particularly strong if it does not need to be tuned for different / unique experimental designs, and the behaviour simply emerges from the model for all conditions.

Based on this, I would encourage that the authors include this specific concern as a limitation within in the discussion. Perhaps, simply stating: “A limitation of our approach is that the cost function was manipulated for each experimental condition, rather than predictions emerging for each condition using a model with a constant set of parameters.”

We understand the Reviewer’s concern, but we must stress that this it is a major point of disagreement. Tasks with a target region that have different structures cannot be reproduced with a single cost-function. It is not an assumption of OFC theory that behaviours must be reproduced with a single instance of a model. All examples of studies on task-dependent (synonymous of cost-function-dependent) feedback control in the sensorimotor system use changes in the cost-function that match experimental conditions. It is the case for passing through 5 or 21 targets (Todorov & Jordan, 2002), for uni- versus bi-manual corrections (Diedrichsen, 2007), for velocity-related terminal constraints (Liu & Todorov, 2007), as well as in the Reviewer’s own work (Poscente et al., 2021), where the timing was hand-tuned resulting effectively in changes in the cost-function. We do believe that this concern is orthogonal to the main idea conveyed by our work, and that it is not specific to our study. A more profound debate on this aspect is perhaps necessary but falls outside of the scope of our work. We nevertheless reworked the limitation section of the manuscript to highlight that caveat of the model (**lines 482-488**, see below):

*“Our approach has some limitations. The cost functions were independently defined for each experimental condition using a different set of parameters, rather than by a constant set of model parameters that could flexibly express multiple cost functions and from which different behaviors could emerge. This limitation is tightly linked to our choice to model the controller with a linear-quadratic-Gaussian regulator, which constraints the cost function to a quadratic function of the state and action, this problem is recurrent in most of the studies that use the Optimal Feedback Control framework to simulate reaching movements* (Diedrichsen & Dowling, 2009; Liu & Todorov, 2007; Poscente et al., 2021; Todorov & Jordan, 2002)*.”*

In our opinion, the comment on the falsifiability also reveals a disagreement between our and the Reviewer’s view: clearly our interpretation was falsifiable. If the decision times were constant across the tested conditions of perturbation amplitude and reward landscape, then our claims would be proven wrong, and the paper would not exist, or would conclude for a different mechanism that does not produce changes in decision time.

2.) *“By introducing the cost-to-go as a decision variable, we bridged an important gap between current models of motor control and decision making. We used the cost-to-go as a decision variable because of its theoretical grounding in the dynamic programming solution of the optimal control problem (54). It has been used to derive locally and globally optimal solutions to the control problem through stochastic optimal control (27,55) and reinforcement learning (56,57). The implementation presented in this paper allows to go beyond standard static formulation of OFC models with fixed parameters, and explicitly integrate the reward, changes in target structure and multiple targets in the cost-to-go, similarly to what was done with the value function (i.e. the opposite of the cost-to-go) in reinforcement learning (58). It is important to mention previous studies investigating how humans performed reaching movements in a paradigm where two or more targets were presented prior to movement, and the correct one was cued later (59–62). The behavior observed in this go-before-you-know paradigm can be reproduced by averaging the control policies or target locations associated with the different options (62–65), simulations not shown. Thus, from a computational perspective this problem does not require to update the controller online which differs from the experimental results that we modelled. In addition, in our task, no target was cued before or during movements, the controller must decide between different valid choices instead of waiting to see which one is correct. However, a controller endowed with dynamic updates based on continuous tracking of the cost-to-go can also handle the problem of reaching to uncertain targets.”*

LATEST COMMENT: The authors have included simulations for a go-before-you-know task in the rebuttal and mentioned them in the discussion. From their response above, it seems that they used a model without continuous monitoring (which they mention is not necessary). They also state in the updated manuscript that a controller with dynamic updates based on continuous tracking of the cost-to-go can handle reaching to uncertain target.

Coming back to the Wong and Haith (2017) paper, a critical experimental condition they have not captured with their rebuttal simulations is the fast condition. Specifically, humans will not select an ‘average’ trajectory when there is a heavy time constraint, but rather select the left or right target by moving directly towards that target (see Figure 2D, bottom row with blue trajectories in the Wong and haith paper). The authors’ model would not make this prediction given its current formulation. It is understood that the focus of the paper is online changes, but it is also important to clearly highlight what aspects of the decision-making / motor control literature that their model can or cannot explain.

Can the authors please make a more direct statement that the model in its current formulation does not capture all of the results in the Wong and Haith (2017) and Alhussein and Smith (2021). Perhaps, “In the current formulation of our model, we are not able to capture all the experimental conditions in the Wong and Haith (2017) or Alhussein and Smith (2021). However, a controller endowed with…”

We reworked the paragraph of the discussion where these studies were mentioned be more transparent concerning the limitations of our model. This was a comment made by several Reviewers so we re-emphasized that section of the discussion. We now explicitly mention that we were able to reproduce these results only partially (see lines **477-481**).

*“In the current version of our model, we are not able to capture all the experimental conditions in the Wong and Haith* (Wong & Haith, 2017) *or Alhussein and Smith* (Alhussein & Smith, 2021) *studies,* ***however a controller performing dynamic updates of its policy based on continuous tracking of the cost-to-go****, such as the one developed in the present study, can also handle the problem of reaching to uncertain targets.”*

As a minor comment, I would remove the word endowed in their currently written sentence and replace with another.

This was corrected (see the bold highlight in the text snippet above), thanks.

Sincerely,

Josh Cashaback

**Reviewer 2**

The authors have done a great job answering every single question of the 3 reviewers. Yet I am still completely confused about the receding horizon. Does the model involve a receding horizon?

lines 95-97
*"We modeled online modifications in control by implementing a receding horizon controller inspired by model predictive control (33,34), which was previously used to study sensorimotor control (35–37)"*

lines 575-576
*"N is considered to be a finite integer which characterizes a finite horizon formulation of the control problem"*
At this stage, I understand that N is given at the beginning of the simulation and is decremented at each time step.

line 584
N is now in the set of task parameters so maybe it means that N can be changed.

line 602-605
*"The basic premise of this implementation is that the set of optimal feedback control gains Lt are computed at each time step from the time-varying set of task parameters theta(t) and only the first motor command is applied, similarly to what is done in the MPC framework. The procedure is then repeated at the next time step, for which Q and N are adapted to respectively capture time-varying task demands and changes in movement horizon. The time horizon (N) was decremented at each time step such that the total movement time remained constant."*
I do not understand. Horizon can be changed during the movement but you manage so that movement time is constant. The logic escapes me.
Note again that the fact that "only the first motor command is applied" is not specific to MPC but is also in the definition of optimal feedback control (Bryson and Ho 1975, p 128).

From wikipedia (https://en.wikipedia.org/wiki/Model_predictive_control)
"MPC is based on iterative, finite-horizon optimization of a plant model. At time t the current plant state is sampled and a cost minimizing control strategy is computed (via a numerical minimization algorithm) for a relatively short time horizon in the future: [t,t+T]. Specifically, an online or on-the-fly calculation is used to explore state trajectories that emanate from the current state and find (via the solution of Euler–Lagrange equations) a cost-minimizing control strategy until time t+T. Only the first step of the control strategy is implemented, then the plant state is sampled again and the calculations are repeated starting from the new current state, yielding a new control and new predicted state path. The prediction horizon keeps being shifted forward and for this reason MPC is also called receding horizon control. Although this approach is not optimal, in practice it has given very good results. Much academic research has been done to find fast methods of solution of Euler–Lagrange type equations, to understand the global stability properties of MPC's local optimization, and in general to improve the MPC method."

Things should not be complicated. There are two approaches (forget the infinite horizon formulation):
1. Fixed horizon
Movement duration T is given in advance. The number of steps is N = T/timestep. The algorithm is the following: for each n in [0,N], optimize over [n,N]. This is the classical optimal feedback control used by Todorov and Jordan.
2. Receding horizon
Simulation (and not movement) duration T is given in advance and a receding horizon TH is defined. The number of simulation steps is N = T/timestep and the number of steps of the horizon is NH = TH/timestep. The algorithm is the following: for each n in [0,N-NH], optimize over [n,n+NH].

There is a third approach that we could call "variable horizon":
Movement duration T is given in advance. The number of steps is N = T/timestep. The algorithm is the following: for each n in [0,N], optimize over [n,N], decide to change N (or not) for some reason (perturbation) and use the new N.

What approach is actually used in the model? This is a fundamental question. When you want to compare baseline and perturbed trials in a motor task, the two types of trial will generally lead to different movement durations. A fixed horizon is not a very reasonable way to address perturbations. An adhoc solution is to choose movement duration based on experimental observations (e.g. Liu and Todorov 2007, J Neurosci 27:9354). A principled (but maybe wrong) approach is to use a receding horizon.

We used a fixed horizon approach to generate all our simulations. Movement duration was selected at the beginning of movement corresponding to standard movement time, and decremented at each time step whatever happens during movement. We apologize for letting such a loose definition of the horizon survive across many edits and completely agree that it should have been made very clear from the beginning. It was clarified in the revised version of the manuscript to explicitly mention that we used a finite horizon formulation. Moreover, the updated discussion contains a limitation paragraph that discusses this point in more detail and suggests potential further theoretical developments to account for a possible modulation of movement time (**lines 488-494**):

*“Another important limitation of our model is related to our choice of time horizon for the controller formulation. Indeed, we chose to use a finite horizon controller across all the experimental paradigms, which induces that our model cannot predict the modulation of movement duration with parameters like reward* (Carland et al., 2019; Shadmehr et al., 2010, 2019; Summerside et al., 2018) *or required accuracy* (Fitts, 1954)*. Modulations of movement duration cannot naturally emerge from our model and the incorporation of updates in movement times requires further developments either by considering a receding horizon* (Rigoux & Guigon, 2012) *or an infinite horizon* (Li et al., 2018; Qian et al., 2013)*.”*

Please find below other updates in the text that explains how we handled the time horizon; they correspond to the snippets highlighted by the Reviewer:

**Lines 95-97**

*“We modeled online modifications in control by implementing a* ***dynamical*** *controller inspired by model predictive control* (Guigon et al., 2019; Lee, 2011)*, which was previously used to study sensorimotor control* (Bashford et al., 2022; Darici & Kuo, 2023; Mehrabi et al., 2017)*.”*

**Lines 594-596**

*“The optimal feedback control framework allows to determine the optimal control policy for a given set of task parameters* $\Theta=\left\{ Q,R,N \right\}$*, capturing respectively the structure of goal target, the impact of motor cost, and the* ***remaining*** ***movement*** ***duration****.”*

**Lines 616-620**

*“The procedure is then repeated at the next time step, for which* $Q\left( t \right)$ *and* $N$*are adapted to respectively capture time-varying task demands and decrease in* ***remaining*** ***movement*** ***duration****. The* ***remaining*** ***movement*** ***duration*** ***(N) was decremented at each time step such that the total movement time remained constant****. This recursive implementation of the optimal feedback gains is schematically represented in Figure 7A, in the more general framework of OFC.”*

Emmanuel Guigon

**Reviewer 3**

I appreciate the revisions made to the manuscript in accordance with all reviewers' comments. Thanks to the additional information on the model and the task, my understanding of the proposed model has improved.

However, I have concerns about the generality of the proposed model and its arguments. It appears they are confined only to the Linear Quadratic Gaussian (LQG) framework and cannot be extended to a more biologically plausible model of the optimal feedback control, as proposed by Todorov in 2002.

The fundamental question of how the brain handles signal-dependent noise is the starting point of this theoretical framework. The flexibility in the variance of ongoing movement can only be explained by the optimal feedback control framework, which is capable of incorporating signal-dependent noise in sensory and motor signals. The proposed algorithm is built on dynamic programming and consists of multiple iterations of feedforward steps for state estimation and backward steps for policy optimization. Importantly, these two processes are not independent in this algorithm. Once the cost-to-go is set, the algorithm must compute the entire optimal sequence of state variables (x_hat(t)) to achieve optimal feedback gain over time (see page 1092 of Todorov's 'Stochastic Optimal Control and Estimation Methods Adapted to the Noise Characteristics of the Sensorimotor System', Neural Computation 2005).

Thus, if there are two task parameters for the cost-to-go (Theta_1 and Theta_2) as shown in Fig 7, each optimal feedback control (OFC) component computes a different feedback policy (L_1, L_2) for the different time series of state variables (x_hat1(t=1:end), x_hat2(t=1:end)). Consequently, if the Cost-to-go switches, for instance from 1 to 2 at time t=t_switch, the optimal policy L_2(t_switch) optimizes the sequence of x_hat2(t_switch) rather than the actual state generated by L_1 (i.e., x_hat1). Therefore, if it exists in the biological system as Todorov predicted, the optimal feedback control system would need to recompute the optimal L_2(t=t_swith:end) and x_2(t=t_swithc:end) with the new constraint of the initial state xhat_2_new(t=t_swich)=x_1(t=tswich), which is different from the original x_hat_2.

We thank the Reviewer for their comment and realized that there was some ambiguity in our model formulation, which was removed in the revised version of the manuscript. The comment above mentioned that *each OFC component computes a feedback policy associated with its specific target for different time series of variables*. Importantly, these policies are computed from the same initial or current (estimated) state, in all cases. This is an important point since the controller does not re-use past values at each time step, which enable dynamical adjustments according to the ongoing action. We changed **Figure 7** such that it now explicitly mentions that both alternatives share the same current estimated state ( $\hat{x}_{t}$), see also **lines 713-715** reported below).

*“To integrate multiple target states or alternative options for the movement, we computed the cost-to-go associated with each of these options based on the current state estimate, similarly to what Nashed and colleagues proposed* (Nashed et al., 2014)*.”*

This is a crucial aspect that computational neuroscientists need to address to demonstrate the validity of the proposed integration of task selection and the optimal feedback model. If they ignore signal-dependent noise, they can follow the conventional LQG framework. In this case, thanks to the separation of the controller and the estimator, they can avoid this problem. The authors chose not to address this pivotal issue by stating, "We indeed chose to present our model without signal dependent noise in the paper for simplicity." Although they claimed to have replicated the results with signal-dependent noise, I remain skeptical about how accurately this model was derived and operated given the aforementioned reason. I also disagree with the authors' argument that oversimplification is a reasonable excuse for glossing over this significant aspect. If this issue is not resolved in this model (it is not mentioned at all), I believe that the contribution of this paper is quite limited.

I strongly recommend that the authors revise all simulations in this paper using a model that incorporates signal-dependent noise, and explicitly state how they address this problem in Figure 7.

We agree that the presence of signal-dependent noise is more biologically plausible model while the model we used to produce the simulation results did not include this. However, we verified that this simplification did not affect our conclusions. To address the Reviewer’s concern, we show that SDN can be added to our developments in a new supplementary material, and reproduced the main important results of our paper in this formalism (**Figure S1**, see below). We decided to keep the main results without signal-dependent noise as our conclusions were not altered and it makes the model more understandable by its simplicity.

Figure S1 – Reproduction of the main results in presence of signal-dependent noise. **A** Average reaching behavior to a square (full line black), rectangle (full line gray), square-to-rectangle (dashed line black), and rectangle-to-square (dashed line gray) targets in presence of a leftward mechanical perturbation. **B** Mean and standard deviation of the lateral deviation induced by the mechanical perturbation for the square (full line black), rectangle (full line gray), square-to-rectangle (dashed line black), and rectangle-to-square (dashed line gray) conditions. **C** Individual reach trajectories in presence of lateral perturbation (schematized by the black arrow) in the decision task where targets with different rewards were presented. Red and blue traces highlight movements reaching the central and right target respectively. The relative reward of each target is captured by their color (darker colors represent less rewarding targets). **D** Representation of the mean of the time at which the decision to switch target occurred as a function of reward difference (larger values correspond to larger relative reward for the central target) .**E** Proportion of trial switching to the lateral target as a function of the reward difference. Large reward differences correspond to a larger reward for the central target compared to the lateral ones

The proposed model is very similar to the existing model of online switching of feedback control policy by the observed target information (target ball’s state ) “Ronsse, Renaud, Kunlin Wei, and Dagmar Sternad. "Optimal control of a hybrid rhythmic-discrete task: the bouncing ball revisited." Journal of Neurophysiology 103.5 (2010): 2482-2493.”.

We apologize for missing this previous study and integrated in the part of the discussion discussing previous work (**lines 422-423**).

While it should be appreciated that the author explained some reported data by their group by using the existing task switching architecture of the conventional OFC model without SDN, the novelty and generality of this paper is very limited.

We respectfully disagree with this statement: we provide evidence that decision, planning, and execution stages in the sensorimotor system are not dissociated, and show that it can be captured in OFC framework in which the cost-to-go serves as a contextual decision variable. In our view, our study contributes to a broader understanding of sensorimotor processes and open novel perspectives such as studying the links between decision-making and motor control in more general dynamical contexts, from both theoretical and experimental perspectives. We are currently performing follow-up experiments with clinical populations to gain better insight into the neural functions that are impaired in the targeted populations, and we are convinced that they provide novel and important insight.
